# The Sense of 1PP-Location Contributes to Shaping the Perceived Self-location Together with the Sense of Body-Location

**DOI:** 10.3389/fpsyg.2017.00370

**Published:** 2017-03-14

**Authors:** Hsu-Chia Huang, Yen-Tung Lee, Wen-Yeo Chen, Caleb Liang

**Affiliations:** ^1^Graduate Institute of Brain and Mind Sciences, National Taiwan UniversityTaipei, Taiwan; ^2^Department of Philosophy, National Taiwan UniversityTaipei, Taiwan

**Keywords:** self-location, body-location, first-person perspective, body ownership, double-body effect

## Abstract

*Self-location*—the sense of where I am in space—provides an experiential anchor for one's interaction with the environment. In the studies of full-body illusions, many researchers have defined self-location solely in terms of *body-location*—the subjective feeling of where my body is. Although this view is useful, there is an issue regarding whether it can fully accommodate the role of *1PP-location*—the sense of where my first-person perspective is located in space. In this study, we investigate self-location by comparing body-location and 1PP-location: using a head-mounted display (HMD) and a stereo camera, the subjects watched their own body standing in front of them and received tactile stimulations. We manipulated their senses of body-location and 1PP-location in three different conditions: the participants standing still (Basic condition), asking them to move forward (Walking condition), and swiftly moving the stereo camera away from their body (Visual condition). In the Walking condition, the participants watched their body moving away from their 1PP. In the Visual condition, the scene seen via the HMD was systematically receding. Our data show that, under different manipulations of movement, the spatial unity between 1PP-location and body-location can be temporarily interrupted. Interestingly, we also observed a “double-body effect.” We further suggest that it is better to consider body-location and 1PP-location as interrelated but distinct factors that jointly support the sense of self-location.

## Introduction

The sense of *self-location* has been regarded as a key component of bodily self-consciousness, especially in the research of full-body illusions (Ehrsson, 2007; Lenggenhager et al., 2007; Blanke and Metzinger, 2009; Serino et al., 2013; Maselli, 2015). How is self-location defined in this research field? As a first approximation, the sense of self-location is the subjective feeling of *where I am* in space (Ionta et al., 2011, p. 363; Blanke, 2012, p. 556; Pfeiffer et al., 2014, p. 4021). This understanding is natural, but can only serve as a starting point for investigation. To step forward, many researchers specify the sense of self-location in terms of the sense of *body-location*—the sense of where my body is. In the study by Lenggenhager et al. (2007, p. 1096), participants watched their virtual body in the front while receiving tactile stimulations on the back. Many of them mislocalized themselves “toward the virtual body” during the synchronous condition (2007, p. 1096). Other studies confirmed the findings using different kind of measures, from “the mental ball dropping test” in Lenggenhager et al. (2009), the proprioceptive drift measurement by Aspell et al. (2009), to the measures of peripersonal space by Noel et al. (2015), etc. In the review by Serino et al. (2013), self-location was defined as “the experience of being a body with a given location within the environment” (2013, p. 1239). Applying virtual reality techniques to study bodily illusions, Maselli and Slater (2014) also depicted self-location as “the experience of the body occupying a given portion of space in the environment” (2014, p. 1). Finally, in the fMRI study by Guterstam et al. (2015), self-location was characterized as “the experience that the body is located somewhere in space” (2015, p. 1416). Overall, this definition identifies self-location with body-location, or at least regards the former as determined by the latter.

To be sure, it is very useful to specify self-location in terms of body-location because it not only prevents the Cartesian chasm between self and body, but also makes the notion of self-location experimentally operational. Still, there is a concern: does this way of understanding do justice to another key factor in the sense of self-location, i.e., *first-person perspective* (1PP)? In this study, we assume that the most relevant aspect of 1PP with regard to self-location is its location. It is via its location that 1PP makes contribution to the sense of self-location. So we will speak about the sense of *1PP-location*—the sense of where my first-person perspective is located in space. Also, in this study both body-location and 1PP-location refer to participants' *subjective experiences* rather than the physical locations of their real body or eyes. Thus, during an out-of-body illusion, a subject could feel his/her body-location to be in a place different from the location of his/her real body (Lenggenhager et al., 2009). Similarly, under experimental manipulations, one's sense of 1PP-location could be separated from where one's eyes are physically located in space.

Most studies of self-location, including those just mentioned above, recognize that 1PP plays an important role in the sense of self-location. In the study by Ehrsson (2007), the participants were stroked on the chest which was blocked from view, and saw the stroking applied to a position slightly below the camera. The participants felt as if they were sitting behind their physical body and were looking at it from the location of their “illusory body” (2007, p. 1048). Notice that, in this study the location of the illusory body was determined by the location of the manipulated 1PP (i.e., the location of the camera; cf. also the chest-stroking case in Lenggenhager et al., 2009). In the fMRI study by Ionta et al. (2011), the participants used a cursor to indicate the direction of their 1PP: they felt that they were either looking upward or looking downwards. In our terms, Ionta et al. defined the direction in terms of the location of 1PP (“From where do I perceive the world,” cf. 2011, p. 363). The results showed that “temporo-parietal junction (TPJ) activity reflected experimental changes in self-location that also depended on the first-person perspective” (2011, p. 363, cf. also p. 370, 371). Thus, Serino et al. (2013) suggested that “*perspective* is not wholly distinct from self-location” (2013, p. 1240, authors' emphasis). Finally, in studying judgments about self-location, Starmans and Bloom (2012) found that “children and adults intuitively think of the self as occupying a physical location within the body, close to the eyes” (2012, p. 317). Bertossa et al. (2008) also suggested that “Human volunteers generally seem to find it easy and natural to locate their center of self, the place ‘I am’ or the I-that-perceives. With considerable consistency, sighted or blind, Western or non-Western, it is placed somewhere near the center of their head” (2008, p. 333). Another study by Alsmith and Longo (2014) found that most self-location judgments pointed to either upper face or upper torso. All of these studies indicate a close connection between self-location and the location of 1PP.

Now, if the role of 1PP-location can be incorporated into the role of body-location, then there is probably no need to include the notion of 1PP-location in the definition of self-location. But, is this indeed the case? Before articulating this issue, we will make a few remarks to clarify our terminology. First, although 1PP often refers to one's visual perspective, there is more to it. Other types of information, such as tactile, proprioceptive, vestibular signals, etc. also contribute to one's egocentric reference frame. On the other hand, in order to make the notion of 1PP experimentally operational, many studies consider 1PP as referring to visual perspective. This is reasonable since vision often plays a dominant role relative to other sensory modalities, which is important in the research of full-body illusions. In this study we will operate with the visual notion of 1PP in our experiments, but will take non-visual information into consideration as well. Second, in a recent review, Maselli (2015) defined visual-perspective as “the point from which visual information from the environment is gathered” (2015, p. S309). She chose the term “visual-perspective” instead of “1PP” to avoid confusion with “first-person visual perspective over the fake body” (2015, p. S309). In this study, we will continue to use “1PP” with this caution in mind.

Both body-location and 1PP-location are maintained and influenced by vision, proprioception, somatosensation, and vestibular information. Both are forms of subjective spatial awareness that usually match and integrated with each other. For example, while watching a live baseball game in a stadium, as I move from the outfield to an infield seat, my sense of body location becomes different and my sense of 1PP-location changes accordingly as well. However, we think that there are at least two reasons suggesting that 1PP-location plays a role in self-location that is distinct from body-location, and that a better characterization of self-location should include both body-location and 1PP-location. First, out-of-body experiences (OBE) have been described as a type of abnormal self-location, characterized by a sense of disembodiment and an experience of looking at one's own body from an elevated and distanced 1PP (Blanke and Mohr, 2005, p. 186; Serino et al., 2013, p. 1243). For example, an OBE subject reported that “she saw her whole body as if she were outside, from an external and superior point of view” (Maillard et al., 2004). Another subject said that “she felt she was floating above it and could view her body and its surroundings from above” (Greyson et al., 2014). Blanke and Mohr said that “During an OBE people seem to be awake and feel that their ‘self,’ or center of awareness, is located outside of the physical body and somewhat elevated. It is from this elevated extrapersonal location that the subjects experience seeing their body and the world” (2005, p. 186). These descriptions clearly suggest that, in the case of OBE, the sense of self-location is dissociated from the sense of body-location and tied to the sense of 1PP-location. If self-location is depicted *only* in terms of body-location, how to characterize OBE would become a problem. Hence, 1PP, more precisely, the location of 1PP, is important for specifying self-location, and its role is not the same as body-location.

To see the second reason, consider what Maselli (2015) calls the *front-stroking* and the *back-stroking* paradigms in the studies of full-body illusions. As mentioned earlier, participants in the front-stroking paradigm felt themselves to be in the location of the unseen illusory body (Ehrsson, 2007) and they experienced ownership of that illusory body (Guterstam and Ehrsson, 2012). Here, it is crucial to note that the location of the illusory body was *determined* by the location of the manipulated 1PP (i.e., the location of the camera), *not* the other way around. In the back-stroking case, participants mislocalized themselves toward the virtual body, and some (but not all) of them also experienced it as their own (Lenggenhager et al., 2007). It is worth emphasizing that the virtual body was seen as located 2 m in front of the subject precisely *because* the camera was positioned 2 m behind the subject. These observations suggest that the role of 1PP-location cannot be replaced by body-location. A better picture of self-location seems to be the following: in both the front-stroking and the back-stroking paradigms, self-location requires interaction between body-location and 1PP-location, and it is likely that body-location and 1PP-location are different factors in the sense of self-location.

If 1PP-location and body-location are not the same, will this provide any support to the dualism between self and body? The answer is negative. In everyday life, we experience ourselves as being in the location from where we can perceive the world. Our sense of self-location seems to lock into the 1PP-location given by ordinary experience. Moreover, this ordinary 1PP-location is not an abstract geometric point. There is a sense of embodiment tied to it: we feel that we have a body in (or in line with) that location, from where we can touch and act upon the world. Hence, recognizing the role of 1PP-location in the sense of self-location will not risk falling into Cartesian dualism. In addition, in our previous study on the “self-touching illusion” (Liang et al., 2015), we observed a *double-body effect*: we manipulated the participant's visual perspective while letting him/her interact with the experimenter, such that the subject was touching someone and being touched at the same time, as well as watching his/her own body in front of him/herself. In the two synchronous full-body conditions, many participants felt not only that “I was brushing my own hand” but also that “It felt that I had two bodies” (2015, p. 3–5, Supplementary Materials). If the double-body effect is a solid phenomenon, it would support that 1PP-location is embodied such that there is no tendency toward dualism.

In this study, we investigate self-location by addressing the following issues: first, can the spatial integration of body-location and 1PP-location be temporarily modified? Second, is it possible for healthy subjects to have the illusory experience of owning two bodies? In most previous studies, including both the back-stroking and the front-stroking paradigms, both body-location and 1PP-location remained still throughout the experimental procedures. In this study, we used a back-stroking set-up and added in various forms of *movement* to study body-location and 1PP-location. We aim to propose a refinement of the current picture that characterizes self-location solely in terms of body-location.

Four experiments were conducted to address the above issues: the participants wore an HMD connected with a stereo camera behind them so that they watched their own body standing in front of them while receiving tactile stimulations. Depending on the experiments, the subjects either stood still (Basic condition), or were instructed to walk straight ahead such that they watched their body moving away from the position of their visual perspective (Walking condition), or the experimenter moved the stereo camera away from the subjects' body such that their visual content was systematically receding (Visual condition). Experiment 1 performed the Basic condition. The goal was to verify whether we could induce a bodily illusion similar to the one reported by Lenggenhager et al. (2007), and the results will provide a basis to compare with the data collected in the other conditions. Experiment 2 carried out the Walking condition to see (1) whether a variant of body-ownership illusion could be induced in this condition, and (2) whether the walking movement may modify the participant's sense of body-location. Experiment 3 conducted the Visual condition in order to test: (1) whether another version of body-ownership illusion could be induced in this set-up, (2) whether moving the stereo camera may influence the participant's sense of 1PP-location, and (3) whether it is possible for healthy subjects to feel as if they have more than one body. Finally, in Experiment 4 we performed the synchronous conditions of all the above three experiments. This would enable us to compare the three major conditions so as to investigate the relationship between body-location and 1PP-location.

By conducting these experiments, we intended to test the following hypotheses: (1) the spatial unity between body-location and 1PP-location can be temporarily interrupted in some experimental conditions; and (2) the illusory experience of owning two bodies can be induced. If both hypotheses were verified, they would show that, first, body-location and 1PP-location are two distinct factors in the sense of self-location, and that a better characterization of self-location should include both body-location and 1PP-location. Second, the double-body effect would support the view that the sense of 1PP-location is essentially embodied. Hence, in recognizing the role of 1PP-location, the worry about dualism will not arise. We will discuss the implications of our experimental results and address the issues raised above.

## Methods

### Participants

All four experiments in this study adopted within-subjects designs. Totally, we recruited 86 healthy volunteers. See Table 1 below for the details of the participants. All participants gave their written consent prior to the experiments. All experiments were conducted in accordance with the Declaration of Helsinki. This study was approved by the Research Ethics Committee of National Taiwan University (NTU-REC: 201501HS009).

**Table 1:** **Overview of experiments**.

**Experiment**	**Description**	**Measures taken**	**Participants**
Experiment 1	Sync. condition	Questionnaire	21 (♂ 10)
		SCR	*M* = 21.95 ± 2.12
	Async. condition	Questionnaire	
		SCR	
Experiment 2	Sync. condition	Questionnaire	20 (♂ 14)
		SCR	*M* = 23.40 ± 4.48
	Async. condition	Questionnaire	
		SCR	
Experiment 3	Sync. condition	Questionnaire	20 (♂ 8)
		SCR	*M* = 22.13 ± 1.89
	Async. condition	Questionnaire	
		SCR	
Experiment 4	Basic condition	Questionnaire	25 (♂ 15)
	Walking condition	Questionnaire	*M* = 22.56 ± 3.44
	Visual condition	Questionnaire	

### Materials and procedures

We used a head mounted display (HMD, Sony HMZ-T1) and a stereo camera (Sony HDR-TD20V) to conduct four experiments. The questionnaires were structured using a Likert scale from “strongly disagree” (−3) to “strongly agree” (+3), and the statements were distributed randomly; they can be divided into the following categories: 1PP-location, body-location, body-ownership, 1PP-location vs. body-location, double-body effect, and positive control (**Table 3**). Since the purpose of Experiment 1 was to compare our results with those of Lenggenhager et al. (2007), we adjusted the questionnaire in the following way: Q5 was reformulated as “It felt as if the body in front of me was mine.” We also removed Q2, Q4, Q6, and Q7 from the questionnaire, and added in two statements about touch referral (see Table 2). We also had a screen-switch machine (ATEN, VM5808H, Taiwan) that can switch between the images taken by the stereo camera and other computer images. It allowed us to present questionnaires on the HMD.

**Table 2:** **The questionnaire statements in Experiment 1**.

1PP-location	Q1. It felt as if the position of my first-person perspective had changed.
Body-location	Q3. It felt as if the location of my body had changed.
Body-ownership	Q5. If felt as if the body in front of me was mine.
1PP-location vs. Body-location	Q8. My first-person perspective seemed to be in the back of my body.
	Q9. It felt as if the position of my first-person perspective and my body were not in the same location.
Double-body effect	Q10. It felt as if I had a body here and also had another body in front of me.
Positive control	Q11. I was being brushed during the experiment.
	Q12. It felt as if I were feeling the touch of the brush in the location where I saw the virtual body touched.
Touch referral	Q13. It felt as if I were feeling the touch caused by the brush touching the virtual body.

*The questionnaires were in Chinese when presented to the participants. Here and in Table 3 we present the English translations*.

**Table 3:** **The questionnaire statements in Experiments 2–4**.

1PP-location	Q1. It felt as if the position of my first-person perspective had changed.
	Q2. It felt as if the position of my first-person perspective had not changed.
Body-location	Q3. It felt as if the location of my body had changed.
	Q4. It felt as if the location of my body had not changed.
Body-ownership	Q5. It felt as if the body on the screen was mine.
1PP-location vs. Body-location	Q6. It felt as if my body left the position of my first-person perspective.
	Q7. It felt as if the position of my first-person perspective left my body.
	Q8. My first-person perspective seemed to be located behind my body.
	Q9. It felt as if the position of my first-person perspective and my body were not in the same location.
Double-body effect	Q10. It felt as if I had a body here and had another body in front of me.
Positive control	Q11. I was being brushed during the experiment.

The skin conductance responses (SCR) were recorded with a Data Acquisition Unit-MP35 (Biopac Systems, Inc. USA). SCR was measured in the synchronous and asynchronous conditions of Experiments 1–3, in which a knife was shown on the HMD scene, then cut toward the participant's physical body. To measure SCR, two single-use foam electrodes (Covidien, Inc., Mansfield, USA) were attached to the lower edge of the participant's right palm on the volar surfaces of the medial phalanges. Data were registered at a sample rate of 200 Hz, and analyzed with the Biopac software AcqKnowledge v. 3.7.7. We identified the amplitude of SCR as the difference between the maximal and minimal values of the responses within 5 s of the threat (Dawson et al., 2007). All subjects were informed beforehand that after the experiment they would orally answer a questionnaire presented on their HMD. They were advised to give their answers spontaneously based on their subjective feeling rather than on reasoning. Those subjects who did not show any SCR amplitude and those who did not pass the positive control (i.e., answered negatively to Q11) were excluded from the analyses. Totally, we excluded the data of three participants, including their SCR and questionnaires. See below for the procedures of each experiment.

### Experiment 1: basic condition (sync. vs. async.)

**Figure 1:**
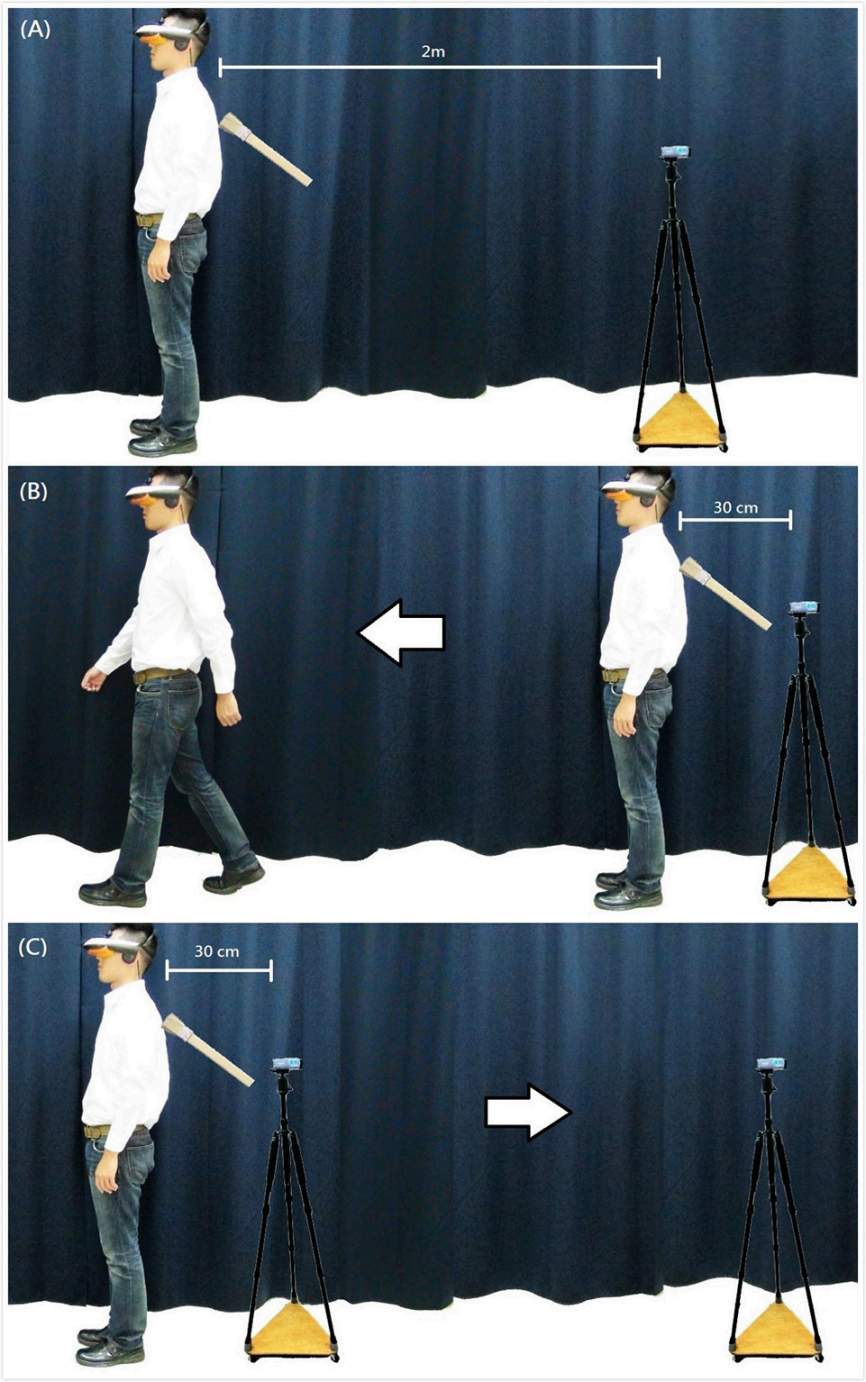
**Experimental set-ups. (A)** Experiment 1 and the Basic condition of Experiment 4. The participant wore an HMD connected with a stereo camera positioned 2 m behind and received tactile stimulations for 70 s. **(B)** Experiment 2 and the Walking condition of Experiment 4. The participant wore an HMD connected with a stereo camera positioned 30 cm behind and received tactile stimulations for 70 s. At the 20th s, the subject was instructed to walk straight ahead for about 2 m. **(C)** Experiment 3 and the Visual condition of Experiment 4. The participant wore an HMD connected with a stereo camera positioned 30 cm behind and received tactile stimulations for 70 s. At the 20th s, the experimenter swiftly moved the stereo camera away from the participant's body for about 2 m.

The participant put on an HMD connected with a stereo camera positioned 2 m behind him/her (Figure 1A). The participant also wore mini-headphones in order to listen to white noise during the experiment. Then the participant was asked to keep his/her eyes closed and wait for the announcement to begin. When the participant opened his/her eyes, he/she saw the back of his/her full body standing in front of him/herself from below the neck. This visual content of the HMD was real-time streaming of the video recording from the stereo camera. The intrinsic delay of the actual streaming was within 20–40 ms. The participant was brushed on the back for 70 s. In the synchronous condition, the visual content matched synchronously with respect to the tactile stimulations. The frequency of the brushing was about once per second. In the asynchronous condition, we played a pre-recorded video on the HMD such that the subject watched his/her back being brushed at a constant speed of about 2 s per stroke. At the same time, the experimenter brushed the participant's back and varied the frequency randomly from 1 to 3 s per stroke, so that the touch that the participant felt was not consistent with what he/she saw. SCR was measured in both conditions at the 60th s: a knife was first shown on the HMD scene for 1 s, then cut toward the participant's upper back (i.e., toward the participant's adopted 3PP) for another 1 s. After the experiment, the participant orally responded to a questionnaire presented on the HMD.

### Experiment 2: walking condition (sync. vs. async.)

The stereo camera was positioned only about 30 cm behind the participant. In the synchronous condition, the participant received synchronous tactile stimulations. At the 20th s, the subject was instructed to walk straight ahead for about 2 m and then was asked to stop (Figure 1B). The average walking velocity was about 0.67 m/s. Since the stereo camera remained in the same position, the walking movement caused changes in the subject's proprioception and visual content: the subject proprioceptively felt that his/her body was moving ahead, while at the same time watching his/her own body moving *away* from his/her visual perspective. The procedure of the asynchronous condition was the same, except that the brushing was asynchronous. In both conditions, the participant received tactile stimulations on the back for 70 s, followed by the same SCR measurement and questionnaires.

### Experiment 3: visual condition (sync. vs. async.)

The stereo camera was again positioned about 30 cm behind the participant, who was brushed on the back either synchronously or asynchronously for 70 s. The new factor was that, at the 20th s, while the subject was standing still, the experimenter swiftly moved the stereo camera away from the subject's body for about 2 m (Figure 1C). The average velocity with which the camera was moved back was about 1.33 m/s. This was to change the location of the participant's 1PP, such that the scene that the subject saw via the HMD systematically receded. The rest of the procedure was the same as in the above two experiments.

### Experiment 4: basic, walking, and visual conditions (sync.)

In this experiment, we conducted the Basic, Walking, and Visual conditions (Figures 1A–C) with only synchronous brushing and did not measure SCR. In each of these conditions, the participant saw via the HMD the back of his/her full body standing in front of him/herself from below the neck, and was brushed on the back for 70 s, followed by a questionnaire.

### Data analyses and statistics

To analyze the questionnaire and SCR data collected in Experiments 1–3, we found that they were not normally distributed (using Shapiro–Wilk tests), so we used the non-parametric Wilcoxon's matched-pairs signed-rank tests to compare the synchronous and asynchronous conditions. For Experiment 4, we conducted Friedman's analyses of variance by ranks to determine whether there were significant differences among the three conditions, followed by Wilcoxon signed-rank tests with Bonferroni correction as *post-hoc* analyses. Wilcoxon signed-rank tests were also carried out to compare Q6 in the Walking and the Basic conditions, and Q7 in the Visual and the Basic conditions. We adopted relatively high standards when interpreting the questionnaire data: in addition to the requirement that differences in data must be statistically significant (α = 0.05), the absolute value of the median of a major factor (such as 1PP-location, body-location, or double-body effect) must be at least one (cf. Kalckert and Ehrsson, 2012). More precisely, if there was an effect on 1PP-location, the median of the positive statement Q1 must be at least positive one (+1), and the median of the negative statement Q2 must be at least negative one (−1). Likewise, if there was an effect on body-location, then Q3 must be at least +1 and the negative statement Q4 must be at least −1. All the other statements were formulated in positive terms, so their median values should reach at least +1 before we claimed to have observed genuine effects. The idea here is that if the absolute value of a median was <1, the group of participants would be considered to be uncertain about the questionnaire statement.

## Results

### Experiment 1

**Figure 2:**
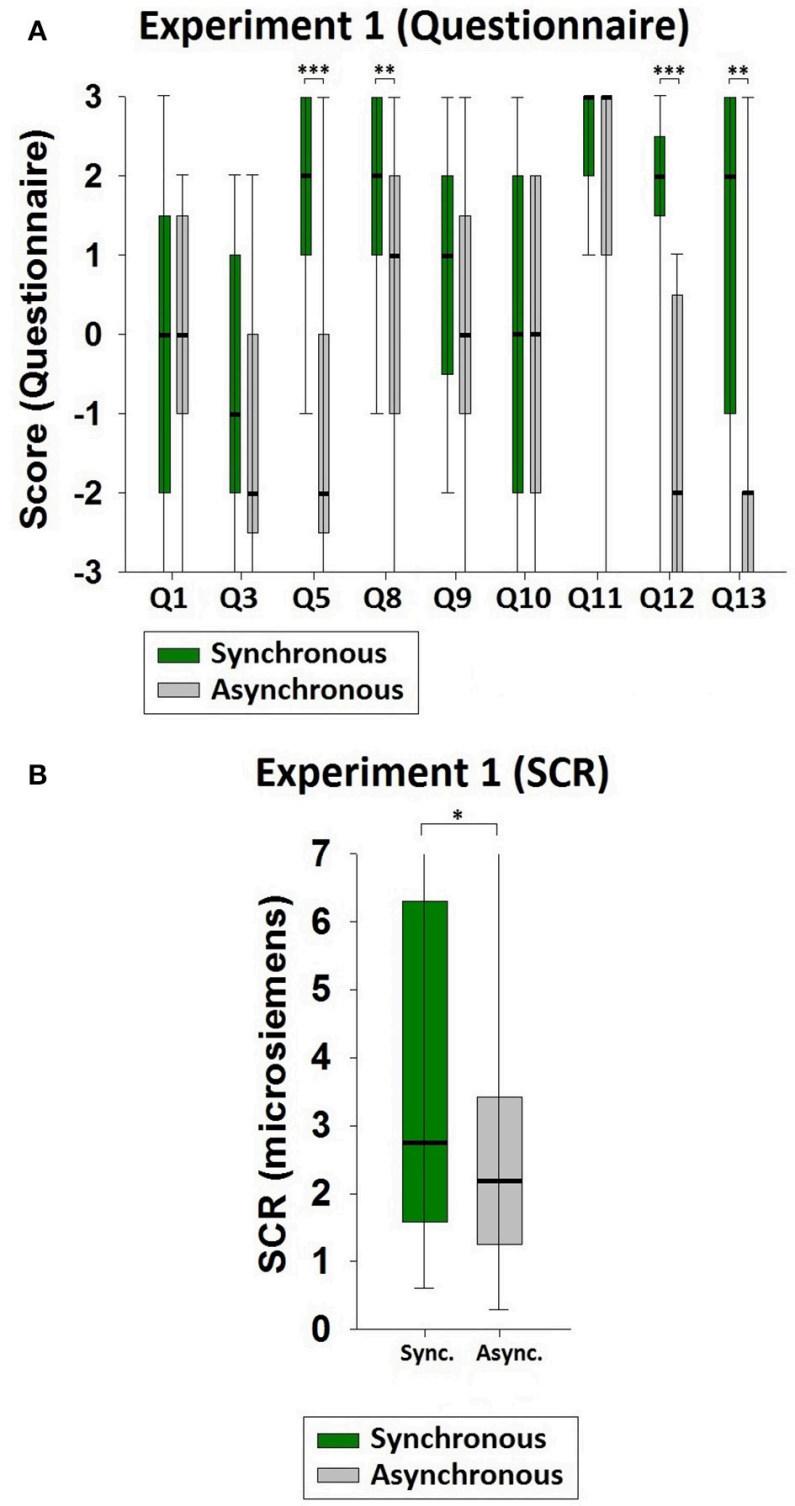
**Results of Experiment 1. (A)** Questionnaire results. There were significant differences between the synchronous and the asynchronous conditions regarding body ownership (Q5), 1PP- location vs. body-location (Q8), and touch referral (Q12 and Q13). **(B)** SCR results. The SCR values were significantly higher in the synchronous than in the asynchronous condition when the knife threats were applied to the participant's physical body (which was viewed via the HMD from the adopted 3PP). Significance levels: ^*^p ≤ 0.05; ^**^p ≤ 0.01; and ^***^p ≤ 0.001.

In this section, we report only the experimental results from significant comparisons. The median values and interquartile ranges (IQRs) of the questionnaire statements of Experiment 1 are shown in Table 4. Statistical significances were observed in Q5 (*z* = −3.662, *p* < 0.001), Q8 (*z* = −2.695, *p* = 0.007), Q12 (*z* = −3.935, *p* < 0.001), and Q13 (*z* = −3.413, *p* = 0.001, Figure 2A). The SCR value was significantly higher in the synchronous than in the asynchronous condition (*z* = −1.964, *p* = 0.050; sync. median = 2.750, async. median = 2.190, Figure 2B). These results suggest that in the synchronous condition the participants felt that their 1PP seemed to be in the back of their body (Q8). More importantly, they felt that the virtual body in front of them was theirs (Q5). The tactile stimulations were felt to be where they saw the virtual body being touched (Q12) and was caused by the brush touching the virtual body (Q13).

**Table 4:** **Median values and interquartile ranges (IQRs) for each question in four experiments**.

**Quest**.	**Experiment 1**		**Experiment 2**		**Experiment 3**		**Experiment 4**		
	**Sync.**	**Async.**	**Sync.**	**Async.**	**Sync.**	**Async.**	**Basic**	**Walking**	**Visual**
Q1	0 (−2, 1.5)	0 (−1, 1.5)	2 (1, 2)	1 (−1.5, 2)	1 (0, 2.75)	0.5 (−1, 2)	1 (−1.5, 1)	1 (−0.5, 2)	2 (1, 2.5)
Q2			−1 (−2, 0.75)	0 (−1, 2)	1 (−1, 2)	1.5 (−1.75, 2)	0 (−1, 2)	1 (−0.5, 2)	−1 (−2, 0)
Q3	−1 (−2, 1)	−2 (−2.5, 0)	2 (−0.75, 2)	1 (−2, 2)	−1 (−2, 1)	−1 (−2, 0)	−2 (−3, 0)	3 (2, 3)	1 (−1, 2)
Q4			−0.5 (−2, 1)	−1 (−1.75, 1.75)	2 (1.25, 3)	2 (2, 3)	2 (1, 3)	−2 (−3, −1)	1 (−0.5, 3)
Q5	2 (1, 3)	−2 (−2.5, 0)	3 (2, 3)	1.5 (−1.75, 3)	3 (2, 3)	0 (−2, 1)	3 (2.5, 3)	3 (2, 3)	3 (2, 3)
Q6			1 (0, 2)	1 (−1.75, 2)			0 (−1.5, 1)	1 (−0.5, 3)	
Q7					0 (−1, 2)	0.5 (−1.75, 2)	0.5 (−2, 1)		2 (1, 3)
Q8	2 (1, 3)	1 (−1, 2)	1 (−0.75, 2)	1 (0, 2)	1 (−1, 2)	0 (−1.75, 2)	1 (0, 2.5)	2 (1, 3)	2 (1, 3)
Q9	1 (−0.5, 2)	0 (−1, 1.5)	1 (0, 2)	2 (−1, 2)	1 (−0.75, 2)	2 (−0.5, 2)	1 (−0.5, 2)	2 (1, 2.5)	2 (1, 3)
Q10	0 (−2, 2)	0 (−2, 2)	1 (−1, 2)	1 (−0.75, 2)	2 (−0.75, 2)	2 (1, 2)	−1 (−2, 2)	1 (0, 2)	2 (1, 2.5)
Q11	3 (2, 3)	3 (1, 3)	3 (2, 3)	3 (3, 3)	3 (3, 3)	3 (2, 3)	3 (3, 3)	3 (3, 3)	3 (3, 3)
Q12	2 (1.5, 2.5)	−2 (−3, 0.5)							
Q13	2 (−1, 3)	−2 (−3, −2)							

*The data marked in red color represent significant comparisons in the experiments*.

### Experiment 2

**Figure 3:**
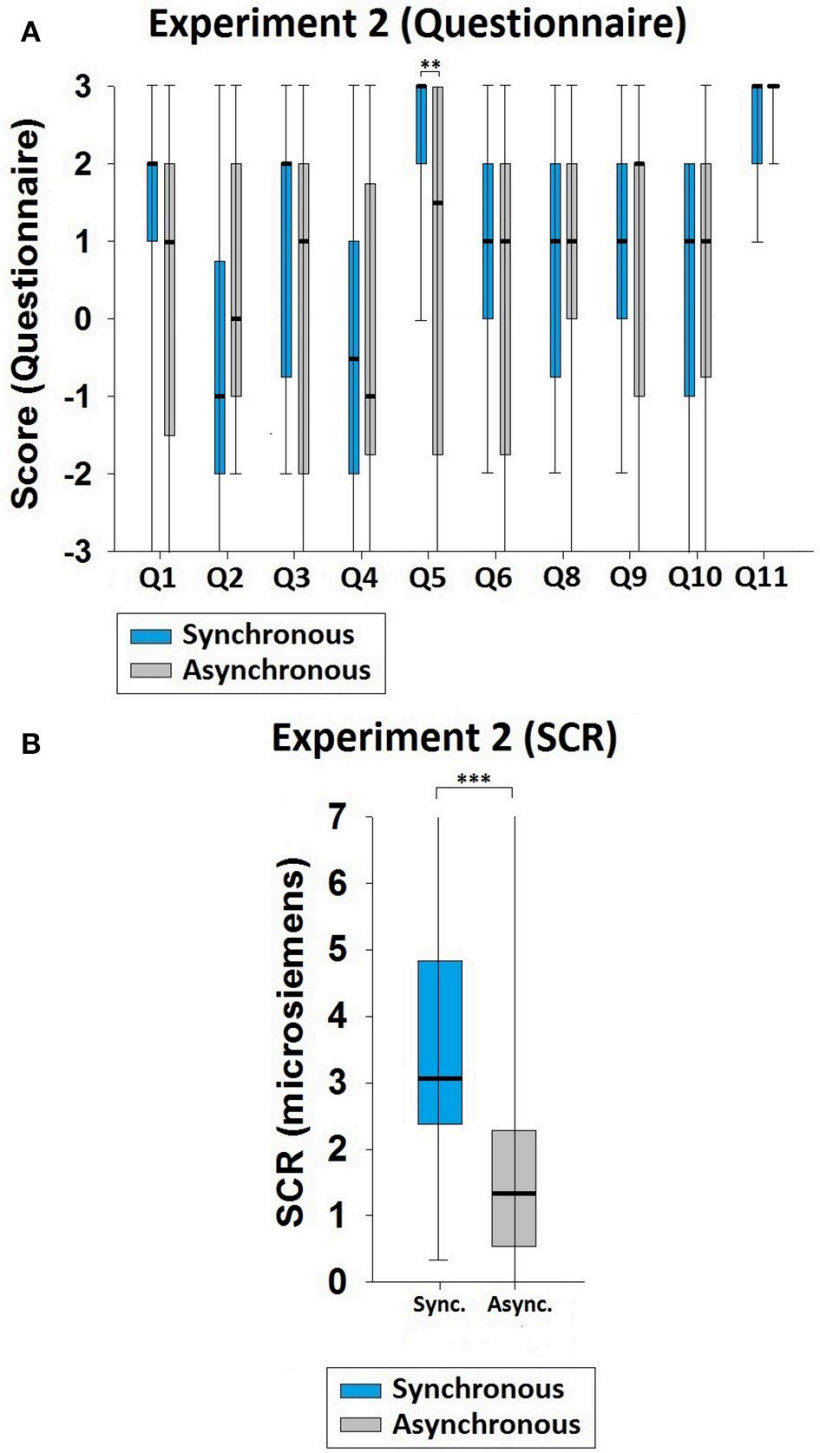
**Results of Experiment 2. (A)** Questionnaire results. A significant difference existed between the synchronous and the asynchronous conditions regarding full-body ownership (Q5). **(B)** SCR results. When the knife threats were applied to the participant's physical body, the SCR values were significantly higher in the synchronous than in the asynchronous condition. Significance levels: ^*^p ≤ 0.05; ^**^p ≤ 0.01; and ^***^p ≤ 0.001.

The median values and IQRs are presented in Table 4. The value of Q5 was significantly higher in the synchronous than in the asynchronous condition (*z* = −2.619, *p* = 0.009, Figure 3A), as was also true for the SCR values (*z* = −3.621, *p* < 0.001; sync. median = 3.061, async. median = 1.342, Figure 3B). The results indicate that, compared with the asynchronous condition, the participants in the synchronous condition experienced ownership of the virtual body in front of them.

### Experiment 3

**Figure 4:**
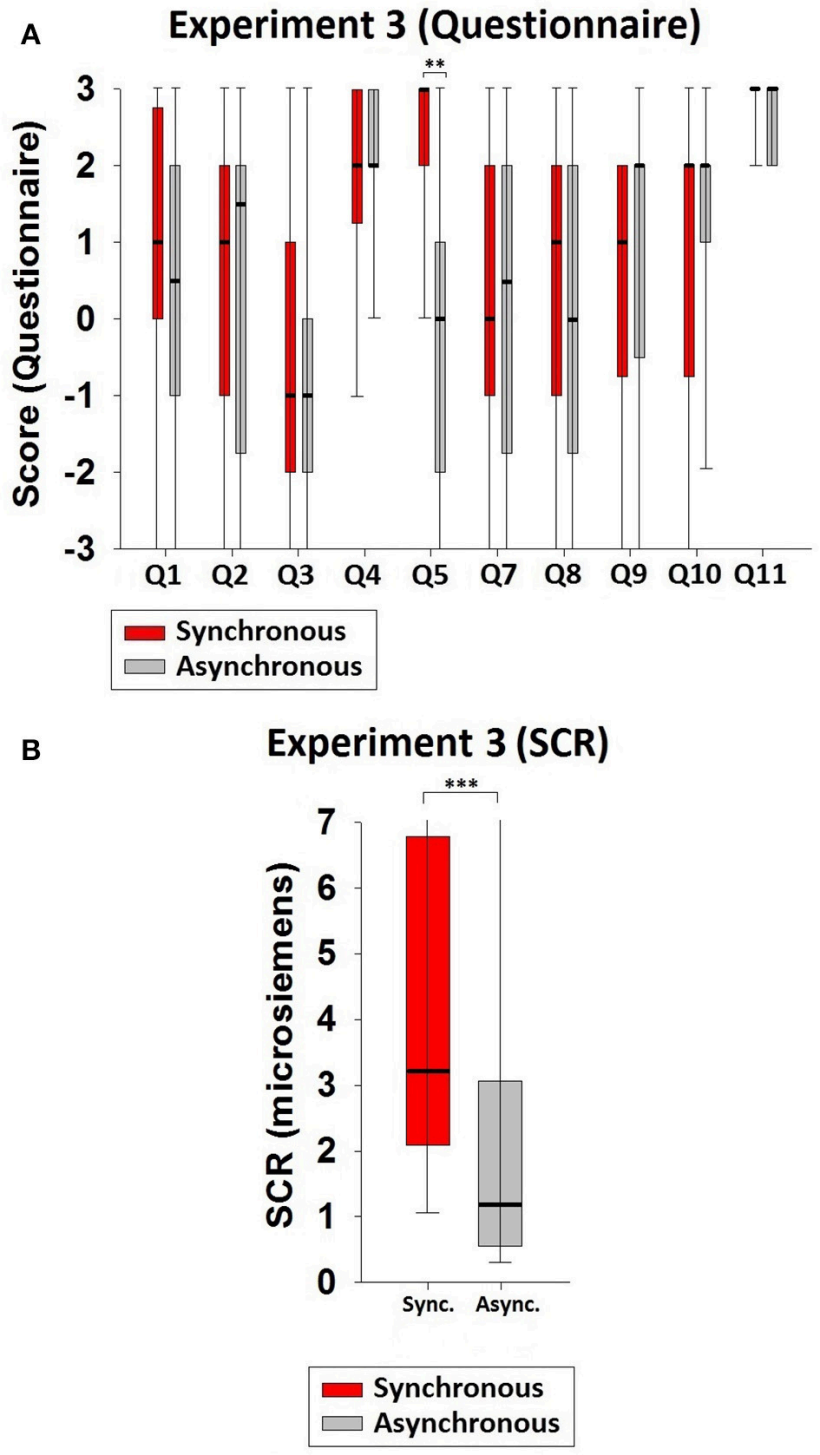
**Results of Experiment 3. (A)** Questionnaire results. A significant difference existed between the synchronous and the asynchronous conditions regarding full-body ownership (Q5). **(B)** SCR results. The SCR values were also significantly higher in the synchronous than in the asynchronous condition when the knife threats were applied to the participant's physical body. Significance levels: ^*^p ≤ 0.05; ^**^p ≤ 0.01; and ^***^p ≤ 0.001.

See Table 4 for the median values and IQRs. The value of Q5 was significantly higher in the synchronous than in the asynchronous condition (*z* = −3.308, *p* = 0.001, Figure 4A), and the SCR values also followed this pattern (*z* = −3.920, *p* < 0.001; sync. median = 3.210, async. median = 1.175, Figure 4B). This also indicates that illusory ownership of the virtual body was induced in the synchronous condition.

### Experiment 4

**Figure 5:**
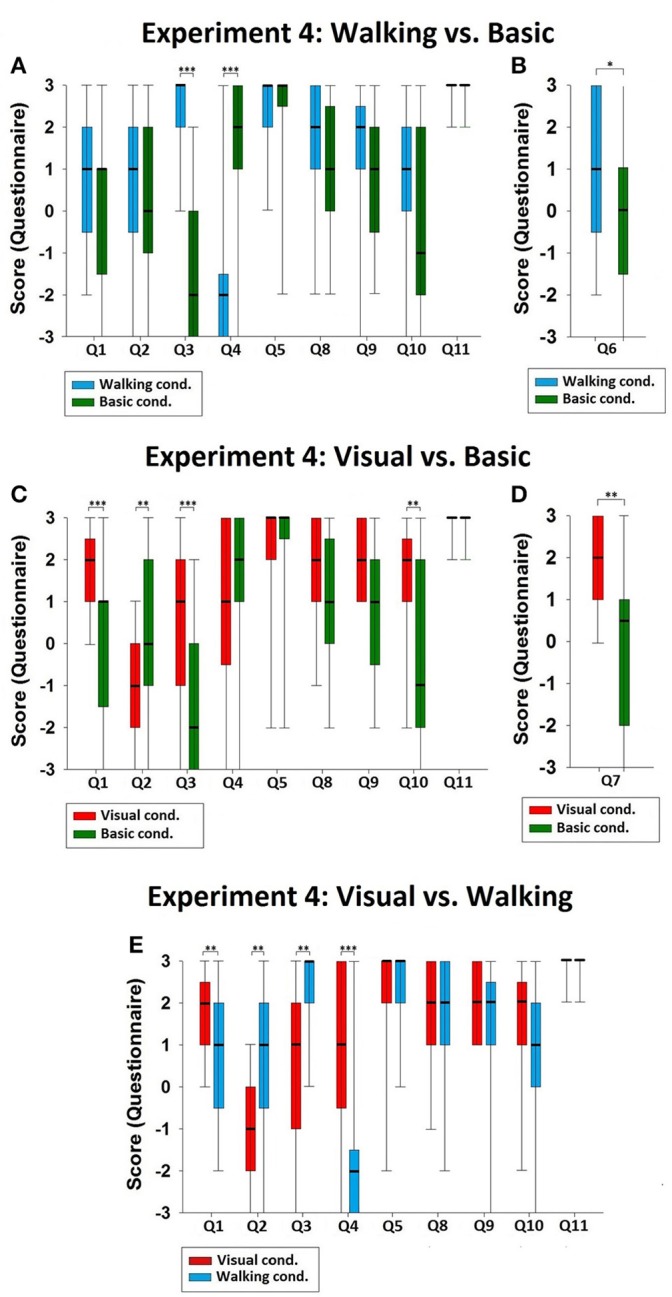
**Comparisons of Experiment 4. (A)** Comparison of the Walking and Basic conditions. The data values of Q3 and Q4 were significantly different, indicating the change in the sense of body-location in the Walking condition. **(B)** Comparison of the Walking and Basic conditions for Q6. The significant difference for Q6 indicated the discrepancy between the sense of body-location and the sense of 1PP-location in the Walking condition. **(C)** Comparison of the Visual and Basic conditions. The significant differences in Q1, Q2, Q3, and Q10 indicated that in the Visual condition there was change in the sense of 1PP-location and the sense of body-location, and that the double-body effect was induced. **(D)** Comparison of the Visual and Basic conditions for Q7. The significant difference for Q7 indicated the discrepancy between 1PP-location and body-location in the Visual condition. **(E)** Comparison of the Visual and Walking conditions. There were significant differences in 1PP-location (Q1 and Q2), and body-location (Q3 and Q4), indicating that the senses of 1PP-location and body-location were distinct between the Visual and the Walking conditions. Significance levels: ^*^p ≤ 0.05; ^**^p ≤ 0.01; and ^***^p ≤ 0.001.

As for the previous experiments, median values and IQRs are presented in Table 4. Using Friedman's analyses, we found that there were significant effects in Q1 (χ^2^ = 16.333, *p* < 0.001), Q2 (χ^2^ = 13.547, *p* = 0.001), Q3 (χ^2^ = 30.644, *p* < 0.001), Q4 (χ^2^ = 23.741, *p* < 0.001), and Q10 (χ^2^ = 6.206, *p* = 0.045). Then we conducted Wilcoxon signed-rank tests with Bonferroni correction (α = 0.05/3 = 0.017). The results are presented in Table 5 (Figures 5A,C,E). Finally, paired Wilcoxon signed-rank tests showed two other significant differences regarding Q6 (Walking vs. Basic: *z* = −2.049, *p* = 0.040, Figure 5B) and Q7 (Visual vs. Basic: *z* = −3.202, *p* = 0.001, Figure 5D).

**Table 5:** **Experiment 4: Paired comparisons of questionnaire scores**.

**Quest**.	**Walking vs. Basic**			**Visual vs. Basic**			**Visual vs. Walking**		
	***z***	***p***	**Cohen's r**	***z***	***p***	**Cohen's r**	***z***	***p***	**Cohen's r**
Q1	−1.469	0.142	0.208	−3.580	<0.001	0.506	−3.225	0.001	0.456
Q2	−0.501	0.617	0.071	−2.875	0.004	0.407	−2.948	0.003	0.417
Q3	−4.135	<0.001	0.585	−3.514	<0.001	0.497	−3.468	0.001	0.490
Q4	−4.106	<0.001	0.581	−1.543	0.123	0.218	−3.582	<0.001	0.507
Q10	−2.026	0.043	0.287	−2.757	0.006	0.390	−1.662	0.096	0.235

*All values were rounded off to the 3rd decimal place. The values in red color represent significant differences. Z-values and p-values are from matched pairs Wilcoxon tests, and effect size is reported by Cohen's r*.

## Discussion

In this study, we investigated self-location by a series of full-body experiments. The findings of Experiment 1 were all consistent with the results reported by Lenggenhager et al. (2007), indicating that we successfully induced a version of out-of-body illusion in the synchronous condition where the participants stood still. In addition to synchronized visual-tactile stimulations, Experiments 2 and 3 brought in different types of movement to induce two different versions of full-body illusion. In the Walking condition, the participants experienced illusory full-body ownership during their walking movement. In the Visual condition, ownership of the virtual body was induced while the participants felt that their 1PP-location was systematically receding. These three experiments provide a good basis for the comparison between body-location and 1PP-location in Experiment 4.

The results of Experiment 4 enable us to address the two issues raised in the Introduction. First, can the spatial unity between body-location and 1PP-location be temporarily modified? Our results have shown that they can. They are different subjective experiences. Compared with the Basic condition, the Walking condition significantly changed the participants' sense of body-location without affecting their sense of 1PP-location, and they felt as if their body left the position of their 1PP (Figures 5A,B). Also, compared with the Basic condition, the Visual condition modulated the sense of 1PP-location such that the participants felt as if their 1PP had left their body (Figures 5C,D). Finally, we observed significant differences between the sense of 1PP-location and the sense of body-location in the comparison between the Visual and the Walking conditions (Figure 5E). These results strongly suggest that the sense of where my 1PP is positioned and the subjective feeling of where I feel my body is located are not the same experiences.

Second, is it possible for healthy subjects to have the illusory experience of owning two bodies? This can be addressed by the data of Experiment 4 about the double-body effect. The score of Q10 in the Visual condition was significantly higher than the Basic condition (Figure 5C, Table 4), indicating that illusory ownership of two bodies is indeed possible. This finding fits well with the report by Lenggenhager et al. (2007) that “None of the participants reported sensations of overt disembodiment” (2007, 1097). Although, the participants felt as if they were watching themselves from a position separated from their body-location, their sense of 1PP-location remained embodied. Hence, given the data on the double-body effect, recognizing the distinct role of 1PP-location in the sense of self-location will not risk falling back to the dualism between self and body.

We think that body-location and 1PP-location are interrelated but distinct factors that jointly support the sense of self-location. Based on our findings, we suggest that, instead of defining self-location only in terms of body-location, the sense of self-location can be better characterized as the subjective experience of *where I am* in space that results from the interaction between body-location and 1PP-location. Below we discuss the implications of our experimental data and compare with other studies.

**(1)** Petkova et al. (2011) argued that viewing the virtual body from 1PP was absolutely crucial for body-ownership illusions to occur. They criticized the 3PP set-up that, since watching the virtual body from 3PP was similar to recognizing oneself on a monitor, the outcome could be just a visual self-recognition “without necessarily experiencing a somatic illusion of ownership” (Petkova et al., 2011, p. 5; cf. also Ehrsson, 2008). In both Lenggenhager et al. (2007) and in our experiments, the subjects watched the virtual body from 3PP via an HMD; hence, both studies would face the above criticism. However, in our Experiments 1–3 we measured the participants' SCR to acquire psychological evidence. Since Lenggenhager et al. (2007) did not do this, our SCR data can be considered as a significant supplement to their pioneering work and can help respond to the above criticism. The significant differences in SCR values between the synchronous and the asynchronous conditions in Experiments 1–3 suggest that the participants' experiences went beyond mere visual self-recognition. Although, there can be alternative interpretations and the issue remains open, the SCR data reported here provide new support for the view that it is possible for 3PP set-ups to induce body-ownership illusions.

**(2)** Our findings about the double-body effect was consistent with the study by Heydrich et al. (2013), where two different methods (an HMD-camera set-up and virtual reality techniques) were used to induce the experience of owning two bodies. Also, as mentioned in the Introduction, our previous study on the “self-touching illusion” also demonstrated that the double-body effect is possible: the subject sat face to face with the experimenter, and both used their right hand to touch each other's left hand with a paintbrush. Under synchronous visual-tactile manipulations, many subjects felt as if they had two bodies (Liang et al., 2015, p. 3–5, Supplementary Materials). So we think that it is possible to induce the double-body effect in healthy subjects.

The set-up of our previous study was similar to the study of body-swap illusion by Petkova and Ehrsson (2008). In one of their experiments (Experiment 5), using visual-tactile manipulations the participant and the experimenter faced each other and squeezed each other's hands synchronously (cf. their Figure 6). Many subjects reported that “I was shaking hands with myself!,” supported by SCR measurements. In another experiment (their Experiment 1), the double-body effect was measured by questionnaire, but no such effect was observed (cf. their Figure 2). Petkova and Ehrsson interpreted these results as showing that the participants felt that their body swapped with someone else's. On the face of it, the body-swap illusion and the double-body effect seem to be different phenomena. Do these experimental results count as against our view? We do not think so. Although, their Experiment 5 involved a subject-experimenter interaction, no questionnaire measurements were conducted and hence the double-body effect was not really tested. In their Experiment 1, the participants only passively received tactile stimulations while viewing a mannequin, and the camera remained still throughout the process (cf. also Petkova et al., 2011). This was very different from the set-up of our current study: compared with the Basic condition, the data of the Visual condition showed that the movement of the camera significantly enhanced the double-body effect. Hence, our view remains sustained that, under the manipulation of moving the camera away from the participants, the experience of owning two bodies could be induced.

**(3)** We have suggested that there is a sense of embodiment associated with the sense of 1PP-location. We would like to further suggest that this sense of embodiment in the 1PP-location is distinct from the sense of 3PP body-location. In both the front-stroking and the back-stroking paradigms, while the participants see their body in front of them via the HMD, the sense of embodiment in the 1PP-location does not rely on viewing the body. In our experiments, the virtual body was seen from the adopted 3PP. The synchronized visual-tactile manipulations caused vision to dominate over tactile sensations and proprioception, such that the illusory sense of self-location was induced. This was consistent with the study by Lenggenhager et al. (2007), in which many participants “mislocalized themselves toward the virtual body” (2007, p. 1096).

In contrast, the sense of embodiment in the 1PP-location is part of everyday experience. We feel that we have a body in (or in line with) the 1PP-location, from where we can perceive, touch, and act upon the world. This sense of embodiment in the 1PP-location is natural and does not depend on seeing one's own body. Moreover, in the Visual condition, the participants' self-location was manipulated by the movement of the stereo camera causing change in the optic flow registered from the 1PP, such that the 1PP-location was felt as if it was receding. Although, the participant stood still, the change in optic flow modified the vestibular sense and elicited an illusory sense of oneself moving backward (illusory self-motion). Previous studies have suggested that vestibular signals can contribute to the sense of self-motion (MacNeilage et al., 2012; Lopez et al., 2013; Barry and Burgess, 2014), and that optic flow can elicit illusory self-motion (DeAngelis and Angelaki, 2012). Also, Lenggenhager and Lopez (2015) suggested that the vestibular system could influence full-body ownership and self-location (2015, p. 17–19). So we think that in the Visual condition the visual 1PP dominated the vestibular signals, such that there is a sense of embodiment tied to the participant's 1PP-location. Therefore, both daily experience and our experimental set-up suggest that the sense of embodiment in the 1PP-location is different from the sense of body-location experienced from the 3PP.

**(4)** In a review article, Blanke (2012) remarks that “In rare instances, however, self-location and first-person perspective can be experienced at different positions, suggesting that it may be possible to experimentally induce similar dissociations in healthy subjects.” Blanke cites the study of OBE by De Ridder et al. (2007) for empirical support, in which a 63-year-old patient was described as follows: “His perception of disembodiment always involved a location about 50 cm behind his body and off to the left…The environment was visually perceived from his real-person perspective, not from the disembodied perspective” (2007, p. 1830). As we see it, two different notions of 1PP were involved in this rare case: the “real-person perspective” and the “disembodied perspective.” The notion of 1PP in Blanke's remark refers to the “real-person perspective,” which was tied to the patient's body-location. What makes this case perplexing was that the patient's sense of self-location split and linked to both the “real-person perspective” and the “disembodied perspective.” Nonetheless, the patient's self-location still involved both the sense of body-location and the sense of 1PP-location in an unusual way, which was compatible with our view.

**(5)** Finally, a very useful account of self-location was recently proposed by Maselli (2015), in which she compared the front-stroking and the back-stroking paradigms. In our terms, this account proposes that in both paradigms self-location is intrinsically connected with and influenced by an embodied 1PP-location, but in very different ways. In the front-stroking paradigm, the experimental manipulation was designed to affect the participant's perceived self-location coded in an allocentric framework. In some studies within this paradigm (Ehrsson, 2007; Guterstam and Ehrsson, 2012), the visual and tactile sensations were both felt in the embodied 1PP-location, such that “the illusory *self-location* corresponds to the position of the *visual-perspective*” (Maselli, 2015, p. S310, author's emphases). In the back-stroking case, the multisensory conflicts can cause a re-coding of the peripersonal space (touch referral) and induce “a spatial dissociation between *visual-perspective* and *self-location*” (Maselli, 2015, p. S310, author's emphases). Thus, Maselli suggests that the sense of self-location can be regarded as “the blending of two parallel representations: the abstract allocentric coding of the position occupied in the environment, mainly associated with the *visual-perspective*, and the egocentric mapping of somatosensory sensations into the external space, mainly associated with *peripersonal space*” (2015, p. S310, author's emphases).

We fully agree with Maselli that both allocentric and egocentric representations are required to account for self-location. We also welcome the emphasis on the role of 1PP in her account. However, there is a difference between her view and ours. Maselli (2015) describes self-location as “the experience of occupying a given position in the environment” (2015, p. S309). This is the natural understanding mentioned in the beginning of the Introduction. But she further characterizes self-location as “the perceived position of the body in space” (2015, p. S309). So she also understands self-location in terms of body-location. As the case of OBE and our experimental results indicated above, we think that it is insufficient to characterize self-location only via body-location. In this regard, our view is different from Maselli's. We propose the following picture: body-location and 1PP-location are two distinct factors that are spatially integrated most of the time, but this integration can be temporarily interrupted in a pathological case or an experimental set-up. Even when the spatial unity of body-location and 1PP-location is temporarily modified, as induced in the back-stroking paradigm, both of these factors continue to interact with each other to maintain an illusory sense of self-location. In our picture, the sense of body-location and the sense of 1PP-location are interrelated factors that jointly support the sense of self-location. On the one hand, both the “3PP body-location” in the back-stroking paradigm and the “illusory body-location” in the front-stroking paradigm are *anchored* in the subject's 1PP-location. On the other hand, 1PP-location is not an abstract geometric point. Rather, it is a subjective experience essentially tied to a sense of embodiment. Self-location results from the interaction between body-location and 1PP-location. If fact, we do not consider our picture to be fundamentally different from Maselli's. However, we do think that when Maselli specifies self-location in terms of the blending of allocentric and egocentric representations, her account is more congenial to our proposal here than construing self-location exclusively in terms of body-location.

## Conclusion

This study investigated self-location by manipulating 1PP-location and body-location. The new methods introduced here—participants' walking movement vs. the displacement of the stereo camera—generated different subjective experiences. Since the sense of self-location is crucial for one's interaction with the environment, we believe that recognizing the distinctive roles of 1PP-location and body-location would contribute to a better picture of environmental adaptation. We would like to make three concluding remarks. First, to situate our study in a broad picture, consider the two different paradigms reviewed by Rosch (2000). One is “analytic science”: according to Rosch, “The analytic picture offered by the cognitive sciences is this: the world consists of separate objects and states of affairs …it deals with isolated units” (2000, p. 189–190). The other is “biofunctionalism”: as Rosch characterizes it, in daily life there is “a powerful intuition of wholeness which goes beyond conceptual analysis into isolated units” (2000, p. 190). As Gibson suggested, “the words animal and environment make an inseparable pair. Each term implies the other” (Gibson, 1979, p. 8). In our experiments, the visual perspective was manipulated such that it felt as if the participant's 1PP was separated from his/her body. This was not an ordinary context. In this sense, we agree that our experiments are within the paradigm of analytic science. So what we have achieved is very modest: we have only demonstrated that the sense of 1PP-location and the sense of body-location can be manipulated selectively in specific settings. We do not claim that our experimental results may automatically apply to ordinary contexts. Second, based on the findings about the double-body effect, we have suggested that 1PP-location is essentially embodied. Hence, both the sense of 1PP-location and the sense of body-location are embodied experiences. We think that both 1PP-location and body-location are inherent in the subjective experience of self-location. The sense of 1PP-location and the sense of body-location jointly contribute to shaping one's experience of self-location. Finally, we would like to suggest an issue for further study. The double-body effect certainly requires further study, and it would be significant to investigate the neural mechanisms that are responsible for self-location as well as the double-body effect. They may help to explain the tremendous flexibility of our bodily experiences in coping with novel environmental challenges. We think that our experiments, especially the Walking and the Visual conditions, could contribute to this endeavor.

## Author contributions

HH and CL designed all experiments; HH, YL, and WC conducted the experiments and analyzed the data; HH and CL wrote the manuscript.

### Conflict of interest statement

The authors declare that the research was conducted in the absence of any commercial or financial relationships that could be construed as a potential conflict of interest.
